# Supplementary material for: The ESRP1-GPR137 axis contributes to intestinal pathogenesis
Source: eLife. 2017 Oct 4;6:e28366. doi: 10.7554/eLife.28366 (PMC5665647; doi:10.7554/eLife.28366)
Supplement: Supplementary file 2. [file elife-28366-supp2.docx]

## Supplementary file 2 – Self-designed primers for qPCR analysis

| Gene | Forward primer 5’->3’ | Reverse primer 5’->3’ |
| --- | --- | --- |
| *Uap1* | TCAGAACGGGAAGGACAACC | GCCTCTGACTTTCCATTTGTAGC |
| *Magi1* | CCTCGGTGCCTGAACAAACA | TCCAGCTCCTCGGTGTGTA |
| *Gpr137_Short* | TGGCTTCTCTATTGCTGCCC | GCTGGTCCCCTGCACAAA |
| *Gpr137_Long* | AGCCTTGCGTACCACACTTT | CTTGAACACCACCTGCACAA |
| *hGPR137_Short* | ACTCCCTGTTCGTCATCTGC | CCTGGCACACACTGGTCC |
